# A partial molecular phylogeny of *Rhadinaea* and related genera (Squamata, Dipsadidae) with comments on the generic assignment of *Rhadinaea eduardoi*

**DOI:** 10.3897/zookeys.943.50738

**Published:** 2020-06-22

**Authors:** Ricardo Palacios-Aguilar, Uri Omar García-Vázquez

**Affiliations:** 1 Museo de Zoología “Alfonso L. Herrera”, Facultad de Ciencias, Universidad Nacional Autónoma de México (UNAM), A.P. 70-399, México D.F., CP 04510, México Universidad Nacional Autónoma de México (UNAM) Mexico Mexico; 2 Posgrado en Ciencias Biológicas, Universidad Nacional Autónoma de México. Circuito de Posgrados, Ciudad Universitaria, Coyoacán, 04510 Ciudad de México, México Universidad Nacional Autónoma de México (UNAM) Mexico Mexico; 3 Laboratorio de Sistemática Molecular, Unidad Multidisciplinaria de Investigación Experimental, Facultad de Estudios Superiores Zaragoza, Universidad Nacional Autónoma de México, Batalla 5 de Mayo s/n, Col. Ejército de Oriente, Ciudad de México 09230, México Universidad Nacional Autónoma de México (UNAM) Mexico Mexico

**Keywords:** *
Coniophanes
*, generic assignment, synonymization, taxonomy

## Abstract

The genus *Rhadinaea* is a diverse clade of New World dipsadid snakes, with 22 species arranged in six recognized species groups. The most recently described species, *Rhadinaea eduardoi*, was described based on a unique specimen collected in the Santa Catarina Juquila municipality in the Sierra Madre del Sur of southern Oaxaca, Mexico. Here, based on a reexamination of the holotype and the results of a phylogenetic analysis of the holotype of *Rhadinaea eduardoi* and representatives of several genera closely related to *Rhadinaea*, we reassessed the generic assignment of *Rhadinaea eduardoi*. In our phylogenetic hypothesis, *R. eduardoi* was nested within a strongly supported clade of *Coniophanes fissidens* samples, thus making *Rhadinaea* paraphyletic with respect to *Coniophanes*. Additionally, our reexamination of the holotype of *Rhadinaea eduardoi* revealed that the alleged presence of a subpreocular scale is only true on the right side of the head, and that this scale appears to be a malformed preocular scale; also, a reduction in dorsal scale rows is present; and posterior enlarged maxillary teeth are grooved. Herein we consider that *Rhadinaea eduardoi* should be placed in the synonymy of *Coniophanes fissidens*. Consequently, we recognized only five species groups within the genus *Rhadinaea*.

## Introduction

Dipsadid snakes are the most speciose family of snakes in the Western Hemisphere, with new species descriptions and taxonomic changes frequently modifying the current composition (García-Vázquez et al. 2018; Mata-Silva et al. 2019). Snakes of the genus *Rhadinaea* Cope, 1863 (Squamata: Dipsadidae) are distributed throughout Mesoamerica, ranging from the Sierra Madre Occidental of southern Sinaloa and Sierra Madre Oriental of northern Nuevo León in Mexico to northwestern Ecuador in South America, with an isolated species, *R. flavilata* (Cope, 1871), in the southeastern USA (García-Vázquez et al. 2018). *Rhadinaea* was formerly considered one of the most diverse New World snake genera, but after several taxonomic changes (see Myers 2011), only 22 species arranged in six species groups are currently recognized. These groups are (number of species in each group in parentheses) the *Rhadinaea calligaster* (1), *R. decorata* (12), *R. eduardoi* (1), *R. flavilata* (2), *R. taeniata* (3) and *R. vermiculaticeps* (3) groups (Myers 1974; García-Vázquez et al. 2018; Mata-Silva et al. 2019). The most recently described species, *Rhadinaea eduardoi* Mata-Silva, Rocha, Ramírez-Bautista, Berriozabal-Islas and Wilson, 2019 is known only from one specimen collected in the municipality of Santa Catarina Juquila in the Sierra Madre del Sur of southern Oaxaca, Mexico. According to the authors, *R. eduardoi* is most closely related to *R. laureata* (Günther, 1868), and is the only representative of its own species group (Mata-Silva et al. 2019). Herein, we present a phylogenetic analysis of *Rhadinaea* and related genera involving species (such as *R. eduardoi*) that were not previously included in the snake phylogeny. Together with a morphological analysis, we use this phylogeny to reassess the taxonomic status of the newly described *R. eduardoi*.

## Materials and methods

### Molecular procedures

To investigate the phylogenetic position of *Rhadinaea eduardoi*, we sequenced a fragment of the mitochondrial gene coding for Cytochrome b (*cyt b*) from 13 individuals including the holotype of *R. eduardoi* (Centro de Investigaciones Biológicas, Universidad Autónoma del Estado de Hidalgo, CIB5457); six samples of the remaining three Mexican species groups of *Rhadinaea*, including *Rhadinaea decorata* (Günther, 1858) (3), *R. taeniata* (Peters, 1863) (2) and *R. laureata* (Günther, 1868) (1); three samples of *Rhadinella*Smith 1941, including one sample each of *R. hempsteadae* (Stuart & Bailey, 1941), *R. lachrymans* (Cope, 1870) and *R. stadelmani* Stuart & Bailey, 1941, previously *Rhadinaea godmani* group; and four samples of *Coniophanes* Hallowell, 1860 (Table 1). Additionally, we obtained sequences from GenBank of an additional sample of *Coniophanes fissidens* (Günther, 1858) and single samples of *Amastridium* Cope, 1860; *Pliocercus* Cope, 1860; *Synophis* Peracca, 1896; and *Tantalophis* Duellman, 1958. All of these genera are considered closely related to *Rhadinaea* by previous authors (Myers 1974, 2011; Pyron et al. 2013). Finally, we used *Hypsiglena jani* Dugès, 1865 to root the tree (Table 1). This region of *cyt b* has been successfully employed to elucidate phylogenetic relationships within Dipsadidae (Lawson et al. 2005; Daza et al. 2009; Pyron et al. 2013). We extracted genomic DNA from muscle or liver tissue using the standard phenol-chloroform method (Hillis et al. 1996), and utilized polymerase chain reaction (PCR) to amplify the aforementioned fragment with the primers L14919, H16064 (Burbrink et al. 2000), L15584 (de Queiroz et al. 2002), and H15716 (Slowinski and Lawson 2002). We sequenced DNA templates with an ABI 3730xl DNA analyzer (Applied Biosystems, Inc.), using primers L14919 and H16064 (Burbrink et al. 2000).

**Table 1.** Collection and voucher data for colubrid genetic samples used in this study. Acronyms for herpetological collections follow Sabaj (2019). RICB, JCSG, OFV, and UOGV are field identifiers for uncatalogued specimens being deposited in the MZFC-HE and UTA.

No.	Voucher number	Taxa	Locality	GenBank accession number
1	CAS228960	*Hypsiglena torquata*	USA: Texas: Culberson Co.	EU728592
2	KU289798	*Coniophanes fissidens* (1)	El Salvador: San Salvador	EF078538
3	RICB521	*Coniophanes fissidens* (2)	Mexico: Chiapas: Road to La Encrucijada	MT308775
4	MZFC-HE34715	*Coniophanes fissidens* (3)	Mexico: Guerrero: Arenal de Gómez	MT308776
5	RICB260	*Coniophanes fissidens* (4)	Mexico: Veracruz: Ocotepec, Los Reyes	MT308777
6	MZFC-HE15533	*Coniophanes imperialis*	Mexico: Oaxaca: Santa Maria Chimalapa, Cofradia	MT308778
7	CIB5457	*Rhadinaea eduardoi*	Mexico: Oaxaca: El Obispo, Santa Catarina Juquila	MT308779
8	UTAR44718	*Rhadinaea decorata* (1)	Guatemala: Huehuetenango: Barillas, Finca Chiblac Buena Vista	MT308780
9	JCSG58	*Rhadinaea decorata* (2)	México: Veracruz: Sierra de Otontepec	MT308781
10	OFV1109	*Rhadinaea decorata* (3)	Mexico: Oaxaca: San Felipe Jalapa de Díaz	MT308782
11	UOGV2181	*Rhadinaea taeniata* (1)	México: Estado de México: Valle de Bravo	MT308787
12	MZFC-HE23859	*Rhadinaea taeniata* (2)	Mexico: Oaxaca Santa Maria Yavesia	MT308788
13	MZFC-HE21661	*Rhadinaea laureata*	México: Morelos: Huitzilac	MT308785
14	UTAR42473	*Rhadinella stadelmani*	Guatemala: Huehuetenango: 3.2 km WSW Patacal	MT308786
15	UTAR42470	*Rhadinella hemsteadae*	Guatemala: Quiche: Uspantán, road El Chimel-San Pablo	MT308783
16	UTAR42335	*Rhadinella lachrymans*	Guatemala: San Marcos: San Rafael Pie de La Cuesta, Finca America El Vergel	MT308784
17	EBUAP1853	*Tantalophis discolor*	México: Oaxaca: Sierra de Monte Flor	EF078589
18	UTAR46905	*Amastridium sapperi*	Guatemala: Izabal	GQ334479
19	QCAZ9175	*Synophis zamora*	Ecuador: Zamora Chinchipe: Las Orquídeas	KT345376

### Phylogenetic relationships

We aligned the obtained sequences using the Muscle algorithm (Edgar 2004) included in the software MEGA 7 (Kumar et al. 2016). The best-fitting substitution models and partitioning schemes were selected jointly using the Bayesian Information Criterion in the software PARTITIONFINDER 1.1.1 (Lanfear et al. 2012). We performed a Bayesian phylogenetic analysis with the software MRBAYES 3.2 (Ronquist et al. 2012). We ran the analysis for 50,000,000 generations with the default settings and tree sampling every 1000 generations. Output parameters were visualized using TRACER 1.4 (Rambaut and Drummond 2007) to verify stationarity and convergence. After discarding the first 25% as burn-in, we summarized parameter values of the samples from the posterior distribution on the maximum clade credibility tree using TREEANNOTATOR 1.4.8 (Drummond and Rambaut 2007) with the posterior probability limit set to 0.1 and mean node heights summarized. We considered clades with posterior probabilities (Pp) ≥ 0.95 as significantly supported (Huelsenbeck and Rannala 2004).

### Genetic distances

To obtain an estimate of genetic distances, we computed pairwise genetic mean distances between *Coniophanes*, *Rhadinaea*, and *R. eduardoi*. We calculated the corrected pairwise genetic distances using the K2P model with MEGA 7 (Kimura 1980; Kumar et al. 2016).

### Morphological comparisons

We compared the holotype of *Rhadinaea eduardoi* (CIB5457) with a series of *Coniophanes* specimens deposited at the Museo de Zoología “Alfonso L. Herrera”, Facultad de Ciencias, Universidad Nacional Autónoma de México (**MZFC-HE**). Scale nomenclature and ventral scale counts follow Myers (1974). To score the dorsal scale rows, we made three separate counts: the first located one head-length posterior to the head, the second located at midbody, and the third located four ventral scales anterior to the anal plate. We counted ventral scales as suggested by Dowling (1951a). Dorsal scale reduction formula is based on Dowling (1951b). Other scutellation characters that we scored were the number of preoculars, postoculars, supralabials, infralabials and subcaudals. We examined the maxillary dentition of the holotype in situ.

## Results

### Phylogenetic relationships

**Figure 1.**
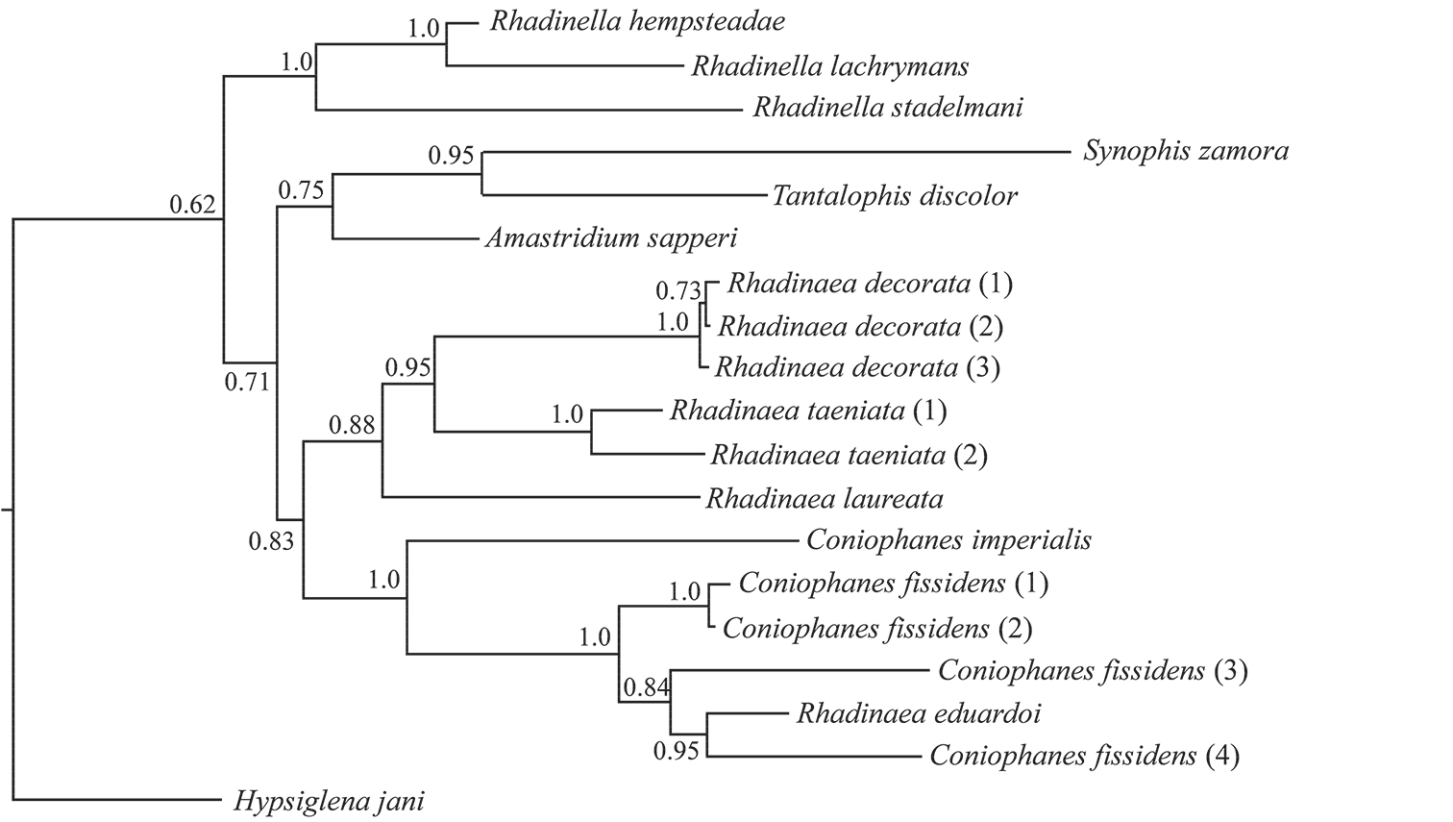
Phylogenetic relationships and phylogenetic position of holotype of *Rhadinaea eduardoi* based on partial sequences of the mitochondrial gene Cytochrome b (*cyt b*). Numbers indicate the Bayesian posterior probabilities for each node.

The final alignment consisted of 1055 bp. The partitions and models that best fit the data were GTR+G for the first and second codon positions, and GTR+G+I for the third codon position. In the Maximum Credibility Tree (Fig. 1), the haplotypes of *Rhadinella* (*R. hempsteadae*, *R. stadelmani*, and *R. lacrymans*) formed the sister taxon to all the remaining haplotypes. Except for the haplotype of *R. eduardoi*, the haplotypes of *Rhadinaea* comprised a clade where *R. decorata* was strongly supported as sister taxon to *R. taeniata* and these two taxa formed the sister taxon to *R. laureata*, the supposedly closest relative of *R. eduardoi* (Mata-Silva et al. 2019), although this relationship was not significantly supported. The *Rhadinaea* clade was the sister taxon to a significantly supported clade comprised of all the haplotypes of *Coniophanes*. The haplotype of *R. eduardoi* was nested within a significantly supported clade composed of all the haplotypes of *C. fissidens*, with *C. imperialis* (Baird & Girard, 1859) as the sister taxon to this clade.

### Genetic distances

Genetic distances between species of *Rhadinaea* and *R. eduardoi* ranged from 18.9–22.4%, whereas distances between species of *Coniophanes* and *R. eduardoi* were much smaller (10.1–11.7%).

### Morphology

*Rhadinaea eduardoi* was originally assigned to *Rhadinaea* due to the presence of a small subpreocular scale, the absence of dorsal scale row reduction, supralabial counts, and dorsal color pattern (Mata-Silva et al. 2019). Our reexamination of the holotype (CIB 5457) verified most of the meristic data presented by Mata-Silva et al. (2019), such as ventral and subcaudal counts. However, two characters differed notably compared to our reexamination: 1) the subpreocular is actually present only on the right side of the head and, furthermore, it appears to be a malformed preocular scale, and 2) dorsal scale row reduction is present, the arrangement being 17-17-15 with counts reduced by fusion of dorsal scale rows 8 + 9, according to the formula:

$17\frac{8+9(75)}{8+9(75)}15(120)$

Of additional diagnostic importance, the maxillary teeth posterior to the diastema are enlarged and grooved. Among other features, the genus *Rhadinaea* is characterized by having a small subpreocular inserted between the corners of two supralabials at the antero-ventral edge of the orbit; the same number of dorsal scale rows throughout the body; and not grooved maxillary teeth posterior to the diastema (Myers 1974, 2011). The characters present in the holotype of *R. eduardoi* are thus inconsistent with the current diagnosis of the genus *Rhadinaea*. Together with the molecular results presented above, this leads us to conclude that the generic allocation of *R. eduardoi* was erroneous.

## Discussion

The phylogenetic relationships obtained in this study are generally consistent with previous phylogenies that suggested a close relationship between *Amastridium*, *Coniophanes*, *Rhadinaea*, and *Tantalophis* (Daza et al. 2009; Pyron et al. 2013), and that supported the separation of *Rhadinella* from *Rhadinaea* (Myers 2011). Pyron et al. (2013) found a strong relationship between *Rhadinaea* and *Coniophanes*, and both with *Tantalophis discolor* (Günther, 1860) and *Amastridium veliferum* Cope, 1860. These relationships are similar to our result, but with the inclusion of *Synophis zamora* Torres-Carvajal, Echevarría, Venegas, Chávez & Camper, 2015 and *Pliocercus elapoides* Cope, 1860 in the same clade of *Amastridium* and *Tantalophis*. Furthermore, we resolved *Rhadinella* as the sister clade of *Rhadinaea* + *Coniophanes*. *Synophis*, *Pliocercus* and *Rhadinella* were not included in the phylogeny of Pyron et al. (2013). Additionally, Daza et al. (2009) found a supported clade formed by *Amastridium sapperi* (Werner, 1903), *Rhadinaea fulvivitis* and *Coniophanes fissidens*, however, *Tantalophis discolor* appear basal to these taxa, plus another dipsadids in an unsupported clade. None of the other genera considered in our study were included by Daza et al. (2009). Although the phylogenetic relationships of *Rhadinaea* with the remaining genera included here were recovered with low support (< 0.95), it is evident that the clade containing *C. fissidens* and *R. eduardoi* is not closely related to the genus (Fig. 1). This result, in addition to the genetic distinctiveness, leads us to consider that the generic allocation of *R. eduardoi* was erroneous. Our revision of morphological characters agrees with this assessment. Hence, we also propose the recognition of only five species groups within *Rhadinaea*.

**Figure 2.**
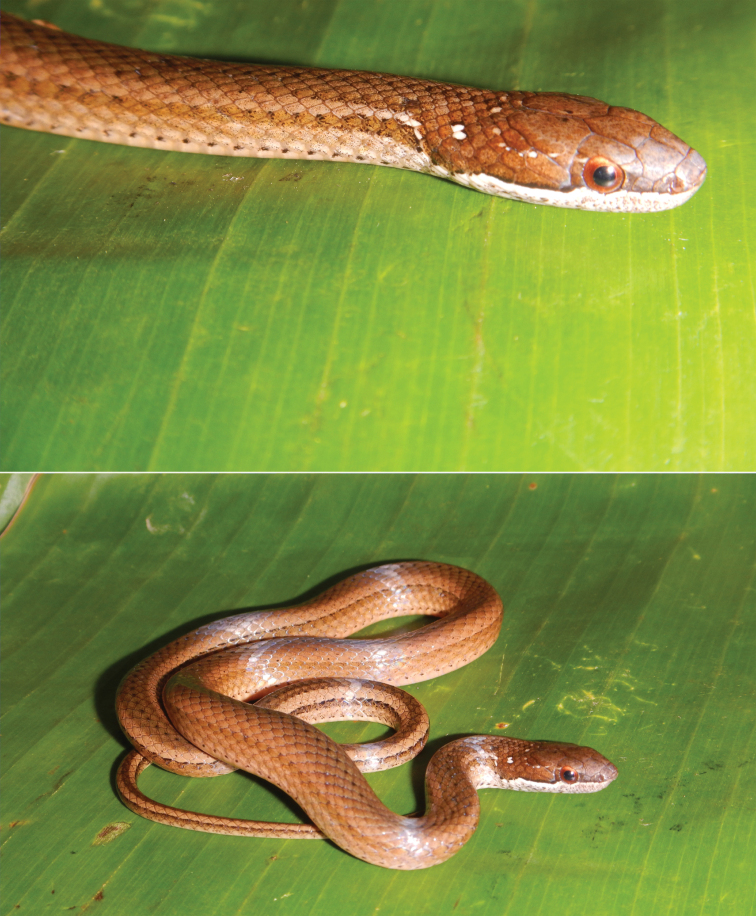
Adult male *Coniophanes fissidens* (MZFC-HE34194) from East of Río Santiago, Guerrero, Mexico. This specimen was obtained approximately 60 kilometers WNW of the type locality of *C. f. dispersus*. Compare this specimen with images 2, 3, and 4 from Mata-Silva et al. (2019).

Bailey (1939) defined the genus *Coniophanes* as consisting of medium sized snakes with enlarged, grooved posterior teeth; posterior dorsal scale reduction through fusion of paravertebral rows; and one or two preocular scales – all characters present on the holotype of *Rhadinaea eduardoi*. The color pattern (diffuse and poorly defined lateral and middorsal stripes on body) and key characters (i.e., posterior dorsal scale reduction by fusion of paravertebral rows; and enlarged, grooved teeth posterior to the diastema) of the holotype clearly allocate it as a representative of *Coniophanes fissidens* (Fig. 2). However, some scutellation characters of the holotype merit discussion. The holotype shows a dorsal scale arrangement in 17-17-15 longitudinal rows, which is very rare in *C. fissidens* (see Smith 1941; and Campbell 1989 for a discussion on the variation exhibited by this species). Of over 100 specimens of *C. fissidens* (Palacios-Aguilar et al. in prep) examined from throughout its range in Mexico, only one specimen (MZFC-HE17791) from Santa María Huatulco, Cuenca del Río Magdalena, Oaxaca exhibited a similar arrangement. The presence of 17 scale rows at midbody is rarely seen in *Coniophanes*, but common in most *Rhadinaea* (Bailey 1939; Myers 1974), likely being one of the factors that led Mata-Silva et al. (2019) to a wrong generic allocation of *R. eduardoi*. The presence of a subpreocular scale is also rare in the genus *Coniophanes*, being consistently present only in the *Coniophanes piceivittis* species group (Bailey 1939; Flores-Villela and Smith 2009). *Coniophanes fissidens* is the most broadly distributed species within the genus, with many subspecies having been described (Smith 1941), and some authors considering it as a species complex (e.g. McCranie 2011).

Based on morphology and geographic distribution, *Rhadinaea eduardoi* is perhaps best considered a junior synonym of *C. f. dispersus*, a subspecies distributed on the Pacific versant of Mexico west of the Isthmus of Tehuantepec from Jalisco to Oaxaca (*sensu* Smith, 1941). However, our phylogenetic tree shows a close relationship between the holotype of *R. eduardoi* and a sample from Veracruz, Mexico (*C. f. fissidens*) which together are the sister clade of a nearly topotypic sample of *C. f. dispersus* (Fig. 1, Table 1). The inclusion of additional samples would help to elucidate this interesting issue. For now, we refrain from recognizing subspecies within *C. fissidens*, pending the acquisition of more samples spanning the species’ wide distribution and the inclusion of additional molecular markers in a more comprehensive study. Hence, we simply suggest the synonymization of *R. eduardoi* with *Coniophanes fissidens* Günther, 1858.

Following the monographic treatment of the genus *Rhadinaea* by Myers (1974), scientific understanding of the composition of the genus has been further modified. The former *brevirostris* and *lateristriga* groups were accommodated in the resurrected genera *Taeniophallus* Cope, 1895 and *Urotheca* Bibron, 1840, respectively (Myers and Cadle 1994). Subsequently, Savage and Crother (1989), and Myers (2011) resurrected *Rhadinella* to include the former *Rhadinaea godmani* group. To date, no large-scale molecular phylogeny has included more than two taxa of *Rhadinaea*, nor any representatives of the genera mentioned above (e.g., Figueroa et al. 2016; Zaher et al. 2019), so the validity of this taxonomy (based only on morphological evidence) remains to be tested in a more comprehensive way. Also, while many authors have agreed that a close relationship between *Rhadinaea* and *Coniophanes* is likely, only a study by Cadle (1984) based on immunological data presented rigorous evidence to support this hypothesis. The present work thus provides the first insights into the phylogenetic relationships of these Neotropical snake genera, supporting the reciprocal monophyly of *Rhadinaea* and *Rhadinella*, and a close relationship between the former genus and *Coniophanes* as sister groups. Only a few morphological characters (dorsal scale reductions, number of preoculars, and teeth grooving) have been considered useful for differentiating *Rhadinaea* and *Coniophanes* (Bailey 1939; Cadle 1989; Myers 1974). As such, additional work including more comprehensive sampling of groups, the use of more molecular markers, and detailed revision of morphology is needed to explore their monophyly and evolutionary history.

## Supplementary materials

Supplementary material 1 Table S1. Specimens examined Data type Specimens examined Explanation note All of the specimens are *Coniophanes fissidens* from Mexico. Acronyms for herpetological collections follow Sabaj (2016). https://binary.pensoft.net/file/424196This dataset is made available under the Open Database License (http://opendatacommons.org/licenses/odbl/1.0/). The Open Database License (ODbL) is a license agreement intended to allow users to freely share, modify, and use this Dataset while maintaining this same freedom for others, provided that the original source and author(s) are credited.Ricardo Palacios-Aguilar, Uri Omar García-Vázquez
