# Supplementary material for: A partial molecular phylogeny of Rhadinaea and related genera (Squamata, Dipsadidae) with comments on the generic assignment of Rhadinaea eduardoi
Source: Zookeys. 2020 Jun 22;943:145–55. doi: 10.3897/zookeys.943.50738 (PMC7324407; doi:10.3897/zookeys.943.50738)
Supplement: Supplementary material 1 — Table S1. Specimens examined [file zookeys-943-145-s001.docx]

**Table S1.** Specimens examined. All of the specimens are *Coniophanes fissidens* from Mexico. Acronyms for herpetological collections follow Sabaj (2016).

| Specimen vouchers | Locality | Latitude (if available) | Longitude (if available) |
| --- | --- | --- | --- |
| CNAR 15113 | Chiapas: Finca San Jerónimo 7.5 km, N Cacaohuatan | – | – |
| CNAR 15114 | Chiapas: Finca San Jerónimo 7.5 km, N Cacaohuatan | – | – |
| CNAR 15115 | Chiapas: Finca San Jerónimo 7.5 km, N Cacaohuatan | – | – |
| CNAR 408 | Chiapas: Santa Rosa, Comitán | – | – |
| CNAR 410 | Chiapas: Santa Rosa, Comitán | – | – |
| CNAR 411 | Chiapas: Santa Rosa, Comitán | – | – |
| MZFC-HE 2088 | Chiapas: 8.5 KM E Estacion Juarez | – | – |
| MZFC-HE 27219 | Chiapas: Rodulfo Figueroa | 16.555389 | -94.18353 |
| MZFC-HE 27220 | Chiapas: Rodulfo Figueroa | 16.555389 | -94.18353 |
| MZFC-HE 28221 | Chiapas: Carretera Catemaco-Dos Amates | 18.48672 | -95.0687 |
| MZFC-HE 28407 | Chiapas: near Las Vegas (N of Escuintla) | – | – |
| MZFC-HE 407 | Chiapas: Tahiti, Island | – | – |
| MZFC-HE 34194 | Guerrero: Arroyo E Río Santiago | 17.26015 | -100.29903 |
| MZFC-HE 17852 | Hidalgo: Lontla | 21.0355 | -98.6388 |
| MZFC-HE 7175 | Hidalgo: 2.5 KM N of desviation to Lontla | – | – |
| MZFC-HE 25934 | Jalisco: La Cumbre, Reserva de la Biósfera Sierra de Manantlán | – | – |
| CNAR 25289 | ND: No data | – | – |
| CNAR 28064 | ND: No data | – | – |
| CNAR 30181 | ND: No data | – | – |
| CNAR 31395 | ND: No data | – | – |
| CNAR 31463 | ND: No data | – | – |
| CIB 5457 | Oaxaca: El Obispo, Santa Catarina Juquila | 16.183573 | -97.305614 |
| CNAR 23847 | Oaxaca: Parque Nacional Lagunas de Chacahua | 15.97433 | -97.6779 |
| CNAR 24759 | Oaxaca: San Felipe Usila, Santiago Tlatepusco | 17.84939 | -96.51086 |
| MZFC-HE 13587 | Oaxaca: 4 km E Las Peñas | – | – |
| MZFC-HE 16961 | Oaxaca: Road San Dionisio del Mar- Chicapa de Castro | 16.41978 | -94.78139 |
| MZFC-HE 17791 | Oaxaca: Santa María Huatulco, Cuenca Río Magdalena | 15.85 | -96.36 |
| MZFC-HE 18110 | Oaxaca: La Victoria | 16.373361 | -95.129027 |
| MZFC-HE 18266 | Oaxaca: Chalchijapa | – | – |
| MZFC-HE 21322 | Oaxaca: San Pedro Ocoltapa | 16.9492 | -95.8325 |
| MZFC-HE 22508 | Oaxaca: Road S Finca El Carmen | 15.88183 | -96.42269 |
| MZFC-HE 22583 | Oaxaca: Finca El Carmen | 15.884583 | -96.379916 |
| MZFC-HE 22629 | Oaxaca: Juquilita river | 15.90245 | -96.453 |
| MZFC-HE 26836 | Oaxaca: Mx 175 between San Pedro Pochutla and Miahuatlán | 16.05703 | -96.50317 |
| CNAR 18632 | Puebla: Hueytamalco, Rancho Las Margaritas | 20.00293 | -97.30255 |
| MZFC-HE 23306 | Puebla: El Canal | 19.95014 | -97.48972 |
| MZFC-HE 23308 | Puebla: Presa La Soledad | 19.95972 | -97.44572 |
| MZFC-HE 24618 | Puebla: 1.3 km NE Lancho | 19.19583 | -97.05466 |
| MZFC-HE 28937 | Puebla: Talcozaman | 19.91121 | -97.63994 |
| MZFC-HE 8861 | Querétaro: San José de Los Paderones | 21.61972 | -99.20194 |
| MZFC-HE 9791 | Querétaro: 2 Km NE Neblinas | – | – |
| MZFC-HE 13901 | San Luis Potosí: San Nicolas de los Montes | 22.125 | -99.4075 |
| CNAR 1512 | Veracruz: Montepío | 18.64 | -95.09 |
| CNAR 20230 | Veracruz: Balzapote | 18.61667 | -95.06278 |
| CNAR 20909 | Veracruz: Estación de Biología Tropical "Los Tuxtlas"UNAM | 18.58513 | -95.0752 |
| CNAR 20910 | Veracruz: Estación de Biología Tropical "Los Tuxtlas"UNAM | 18.58513 | -95.0752 |
| CNAR 20912 | Veracruz: San Andrés Tuxtla | – | – |
| CNAR 20913 | Veracruz: Estación de Biología A Laguna Escondida | 18.59 | -95.08111 |
| CNAR 20916 | Veracruz: Estación de Biología Tropical "Los Tuxtlas"UNAM | 18.58513 | -95.0752 |
| CNAR 20917 | Veracruz: 1.5 KM of Balzapote | 18.58861 | -95.06694 |
| CNAR 20919 | Veracruz: Laguna Escondida | 18.59087 | -95.08827 |
| CNAR 20922 | Veracruz: Estación de Biología Tropical "Los Tuxtlas"UNAM | 18.58513 | -95.0752 |
| CNAR 20923 | Veracruz: Estación de Biología Tropical "Los Tuxtlas"UNAM | – | – |
| CNAR 20924 | Veracruz: Estación de Biología Tropical "Los Tuxtlas"UNAM | 18.58513 | -95.0752 |
| CNAR 20925 | Veracruz: Cuauhtemoc, Volcán San Martín | 18.48778 | -95.10417 |
| CNAR 20927 | Veracruz: Estación de Biología Tropical "Los Tuxtlas"UNAM | 18.58513 | -95.0752 |
| CNAR 20930 | Veracruz: Laguna Escondida | 18.59087 | -95.08827 |
| CNAR 20931 | Veracruz: Laguna Escondida | 18.59087 | -95.08827 |
| CNAR 20932 | Veracruz: Selva, Estación de Biología | 18.57528 | -95.08 |
| CNAR 20933 | Veracruz: Estación de Biología Tropical "Los Tuxtlas"UNAM | 18.58513 | -95.0752 |
| CNAR 20934 | Veracruz: Ocotal Chico | 18.25111 | -94.84028 |
| CNAR 20935 | Veracruz: San Andrés Tuxtla | – | – |
| CNAR 20938 | Veracruz: Laguna Zacatal | 18.58361 | -95.10639 |
| CNAR 20939 | Veracruz: Selva, Estación de Biología | 18.57528 | -95.08 |
| CNAR 20940 | Veracruz: Estación de Biología Tropical "Los Tuxtlas"UNAM | 18.58513 | -95.0752 |
| CNAR 20941 | Veracruz: Selva, Estación de Biología | 18.57528 | -95.08 |
| CNAR 20942 | Veracruz: Estación de Biología Tropical "Los Tuxtlas"UNAM | 18.58513 | -95.0752 |
| CNAR 20948 | Veracruz: Laguna Escondida | 18.59087 | -95.08827 |
| CNAR 20949 | Veracruz: Estación de Biología Tropical "Los Tuxtlas"UNAM | 18.58513 | -95.0752 |
| CNAR 20950 | Veracruz: Estación de Biología Tropical "Los Tuxtlas"UNAM | – | – |
| CNAR 20953 | Veracruz: San Andrés Tuxtla | – | – |
| CNAR 20955 | Veracruz: Estación de Biología Tropical "Los Tuxtlas"UNAM | 18.58513 | -95.0752 |
| CNAR 20956 | Veracruz: Laguna Escondida | 18.59087 | -95.08827 |
| CNAR 20957 | Veracruz: Selva, Estación de Biología | 18.57528 | -95.08 |
| CNAR 20959 | Veracruz: Estación de Biología Tropical "Los Tuxtlas"UNAM | 18.58513 | -95.0752 |
| CNAR 20960 | Veracruz: Estación de Biología Tropical "Los Tuxtlas"UNAM | 18.58513 | -95.0752 |
| CNAR 20961 | Veracruz: Estación de Biología Tropical "Los Tuxtlas"UNAM | 18.58513 | -95.0752 |
| CNAR 20962 | Veracruz: Estación de Biología Tropical "Los Tuxtlas"UNAM | 18.58513 | -95.0752 |
| CNAR 20965 | Veracruz: Primero de mayo, Volcan San Martín Tuxtla | 18.55694 | -95.19861 |
| CNAR 20966 | Veracruz: Laguna Escondida | 18.59087 | -95.08827 |
| CNAR 20970 | Veracruz: Laguna Zacatal | 18.58361 | -95.10639 |
| CNAR 20973 | Veracruz: Selva, Estación de Biología | 18.57528 | -95.08 |
| CNAR 20974 | Veracruz: Laguna Escondida | 18.59087 | -95.08827 |
| CNAR 20975 | Veracruz: Selva, Estación de Biología | 18.57528 | -95.08 |
| CNAR 20976 | Veracruz: Estación de Biología Tropical "Los Tuxtlas"; UNAM | 18.58513 | -95.0752 |
| CNAR 20977 | Veracruz: Estación de Biología Tropical "Los Tuxtlas"; UNAM | 18.58513 | -95.0752 |
| CNAR 23192 | Veracruz: ND | – | – |
| CNAR 23742 | Veracruz: Coatzacoalcos, Rincón Grande | 18.11672 | -94.31671 |
| CNAR 23770 | Veracruz: Coatzacoalcos, Francisco Villa | 18.13642 | -94.28861 |
| CNAR 23771 | Veracruz: Coatzacoalcos, Francisco Villa | 18.13642 | -94.28861 |
| CNAR 23772 | Veracruz: No data | – | – |
| CNAR 23776 | Veracruz: Coatzacoalcos, Rincón Grande | 18.11672 | -94.31671 |
| CNAR 23778 | Veracruz: Coatzacoalcos, Francisco Villa | 18.13642 | -94.28861 |
| CNAR 23780 | Veracruz: Coatzacoalcos, Francisco Villa | 18.12158 | -94.32817 |
| CNAR 23782 | Veracruz: Coatzacoalcos, Francisco Villa | 18.13336 | -94.28339 |
| CNAR 23784 | Veracruz: Coatzacoalcos, Parque Jaguaroundi | 18.10378 | -94.33617 |
| CNAR 23786 | Veracruz: Coatzacoalcos, Rincón Grande | 18.11672 | -94.31671 |
| CNAR 24161 | Veracruz: Coatzacoalcos, Parque Jaguaroundi | 18.10836 | -94.36364 |
| CNAR 24169 | Veracruz: Coatzacoalcos, Parque Jaguaroundi | 18.10333 | -94.33636 |
| CNAR 25080 | Veracruz: Rancho Los Ramos | 18.58639 | -95.1175 |
| CNAR 27149 | Veracruz: Estación de Biología Tropical "Los Tuxtlas", UNAM | 18.58513 | -95.0752 |
| MZFC-HE 10627 | Veracruz: 12.3 Km ENE Tapalapan, San Martín Volcano | 18.5485 | -95.2363 |
| MZFC-HE 17143 | Veracruz: Falda Volcán Santa Marta: El Bostonal |  |  |
| MZFC-HE 27757 | Veracruz: Villa Nueva | 19.766472 | -96.863999 |
| MZFC-HE 4310 | Veracruz: Bastonal | – | – |
| MZFC-HE 4662 | Veracruz: Bastonal | – | – |
| MZFC-HE 8299 | Veracruz: Cerro Tecolayo, Congregación Tapaluca | – | – |
| CIB 5457 | Oaxaca: Santa Catarina Juquila, El Obispo ((holotype *Rhadinaea eduardoi*) | 16.183573 | -97.305614 |
